# Tubeless Retroperitoneal Robot-assisted Partial Nephrectomy: An Innovative Approach to Treat Early Renal Tumor

**DOI:** 10.1590/S1677-5538.IBJU.2026.0042

**Published:** 2026-04-25

**Authors:** Haitao Liang, Yixin Xu, Yulu Peng, Tingxuan Huang, Qiuyue Zheng, Xiangyun Yang, Yunlin Ye, Yanling Liu, Jie Zhou, Zhiling Zhang, Zhenhua Liu, Xia Zheng, Renchun Lai, Pei Dong

**Affiliations:** 1 Sun Yat-sen University Cancer Center State Key Laboratory of Oncology in South China Department of Urology Oncology Guangzhou China Department of Urology Oncology, Sun Yat-sen University Cancer Center; State Key Laboratory of Oncology in South China, Collaborative Innovation Center of Cancer Medicine, Guangzhou, China; 2 Sun Yat-sen University Cancer Center, tate Key Laboratory of Oncology in South China Department of Operating Room Guangzhou China Department of Operating Room, Sun Yat-sen University Cancer Center, State Key Laboratory of Oncology in South China, Collaborative Innovation Center for Cancer Medicine, Guangzhou, China; 3 Sun Yat-sen University Cancer Center State Key Laboratory of Oncology in South China Department of Anesthesiology Guangzhou China Department of Anesthesiology, Sun Yat-sen University Cancer Center, State Key Laboratory of Oncology in South China, Collaborative Innovation Center for Cancer Medicine, Guangzhou, China

**Keywords:** Robotic Surgical Procedures, Kidney Neoplasms, Nephrectomy

## Abstract

**Purpose:**

Retroperitoneal robot-assisted partial nephrectomy (RRPN) has achieved widespread acceptance worldwide. However, the complications and discomfort associated with tubes cannot be overlooked. Consequently, we have initiated Tubeless RRPN (TLRRPN). This approach eliminates the need for tracheal intubation, central venous catheterization, urinary catheters, or abdominal drainage.

**Materials and Methods:**

From 2024-07 to 2025-06, a total of 78 patients underwent RRPN at our center. Following a 1:1 propensity scores matching process, we compared 14 patients who underwent TLRRPN with 14 matched patients receiving Traditional RRPN (TRRPN). Anesthetic management and surgical procedures were systematically detailed, and perioperative outcomes were comprehensively assessed.

**Results:**

All 28 patients underwent successful R0 tumor resection, with no conversions or major complications reported in either group. Patients who received TLRRPN resumed oral intake and ambulation significantly earlier (1.03 hours vs. 22.20 hours and 1.03 hours vs. 30.55 hours, p < 0.001), experienced a shorter postoperative hospital stay (26.00 hours vs. 91.43 hours, p < 0.001), and incurred lower overall costs. No anesthesia- or surgery-related complications were observed, and postoperative pain was significantly reduced in the TLRRPN group compared to the TRRPN group.

**Conclusions:**

TLRRPN is a safe, efficient, innovative, cost-effective, and selective surgical procedure. This operation fundamentally reduces the adverse effects associated with tubes in patients and significantly facilitates rapid recovery.

## INTRODUCTION

In recent decades, robot-assisted partial nephrectomy (RPN) has become increasingly favored due to its advantages of reduced postoperative pain, shorter hospitalization, and faster return to daily activities, while maintaining oncologic outcomes comparable to open partial nephrectomy (OPN) (1-3). The retroperitoneal RPN (RRPN) offers additional benefits by minimizing disturbance to intraperitoneal organs (4-7). Despite these advances, perioperative management often still involves substantial use of sedatives, muscle relaxants, and other pharmacological agents, which may impede postoperative recovery. Moreover, the routine use of tracheal intubation, urinary catheters, drainage tubes, and central venous access can contribute to increased patient discomfort and delayed rehabilitation (8-19). With ongoing progress toward precision surgery and enhanced recovery protocols, a critical question arises: Can RRPN be re-engineered to resemble the minimally invasive, rapid-recovery experience of painless gastroscopy? To explore this concept, our center developed a novel technique—tubeless retroperitoneal robot-assisted partial nephrectomy (TLRRPN)—aimed at eliminating unnecessary perioperative tubing and thereby promoting a truly minimally invasive surgical experience with enhanced postoperative recovery.

TLRRPN—a minimally invasive surgical approach that eliminates the need for tracheal intubation, central venous catheterization, urinary catheterization, and surgical drainage tubes—fundamentally avoids the complications associated with these invasive devices to promote postoperative recovery in patients with early-stage renal tumors.

## MATERIALS AND METHODS

### Surgeon and patient selection

**Figure 1:**
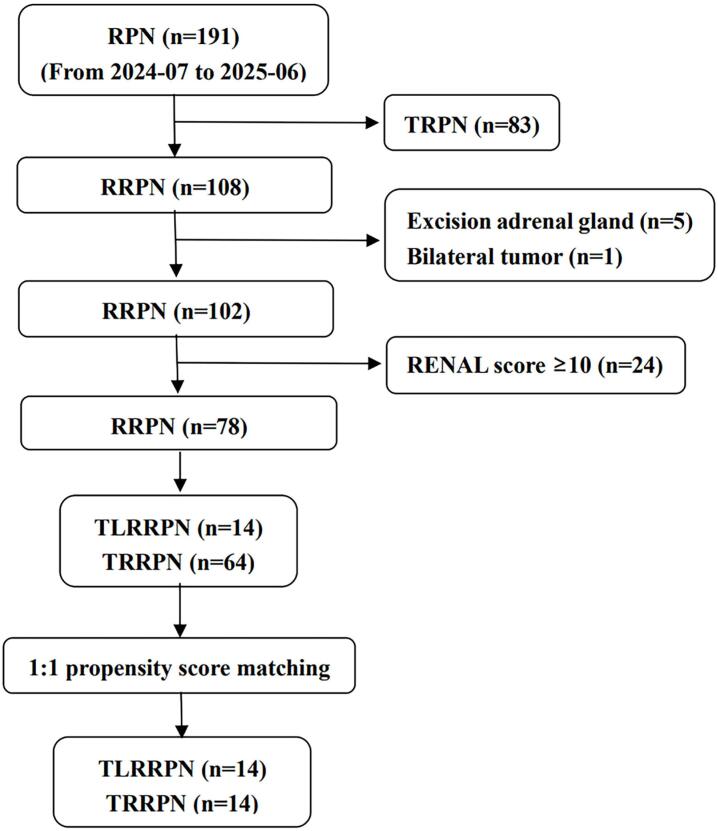
Flow chart of patient inclusion. RPN, robot-assisted partial nephrectomy; TRPN, transperitoneal robot-assisted partial nephrectomy; RRPN, retroperitoneal robot-assisted partial nephrectomy; RENAL, R (radius), E (exophytic/endophytic), N (nearness), A (anterior), L (location); TLRRPN, tubeless retroperitoneal robot-assisted partial nephrectomy; TRRPN, traditional retroperitoneal robot-assisted partial nephrectomy.

Robot-assisted renal surgery has been routinely performed at our center since 2016. All procedures in this study were conducted by a single high-volume surgeon with experience in over 700 robot-assisted nephrectomies. This study has been approved by the institutional review board (IRB) and the ethical committee of Sun Yat-sen University Cancer Center, the IRB number is SL-XJS2025-012. We retrospectively analyzed 78 patients who underwent RRPN between July 2024 and June 2025 (Supplementary Table). Following a 1:1 propensity score matching (PSM), 28 patients were selected and divided into two groups: the TLRRPN (n = 14) and traditional RRPN (TRRPN) groups (n = 14) (Figure-1).

We have frequently conducted TRRPN surgeries in the past. With the enhancement of our proficiency, we initiated TLRRPN in April 2025. We assessed all patients eligible for TLRRPN based on specific criteria: estimated overall operative time ≤ 90 minutes, American Society of Anesthesiologists (ASA) score ≤ 3, RENAL score ≤ 9, absence of cardiopulmonary dysfunction, no history of lower urinary tract obstruction, no significant coagulopathy, and no evidence of gastroesophageal reflux or extrarenal invasion/metastasis. Exclusion criteria were bilateral tumors, concomitant organ resections, and RENAL score ≥ 10 (Figure-1). Baseline characteristics (age, sex, body mass index [BMI], tumor details, and RENAL and ASA scores) and perioperative outcomes were collected. Postoperative recovery time was calculated from the moment of anesthesia awakening.

Follow-up was conducted via telephone interview and review of medical records in accordance with standard clinical guidelines. Patient symptoms were evaluated within 24 hours postoperatively, and complications were evaluated up to 6 months after surgery. The final follow-up was completed on January 1, 2026.

### Preoperative preparation

All patients underwent standardized preoperative evaluation, including contrast-enhanced computed tomography (CT). The surgical site was marked one day before the operation. Bowel preparation was not performed. Patients were instructed to fast from 22:00 on the night prior to surgery. Two units of packed red blood cells were cross-matched and reserved in case intraoperative transfusion was required.

### Anesthetic management

Standard intraoperative monitoring included electrocardiography (ECG), pulse oximetry (SpO_2_), non-invasive blood pressure (NIBP), End-Tidal Carbon Dioxide (ETCO_2_), and Narcotrend depth of anesthesia monitoring.

In the TRRPN group, patients underwent central venous and radial arterial cannulation. General anesthesia was induced and maintained with ciprofol, sufentanil, and rocuronium, targeting a Narcotrend index of 45-60. Ventilation was managed using a single-lumen endotracheal tube under intermittent positive pressure ventilation with 50% inspired oxygen, tidal volume of 6-8 mL/kg, respiratory rate of 12-15 breaths/min, and oxygen flow of 2 L/min.

In the TLRRPN group, ultrasound-guided epidural catheterization (T9/10 or T10/11) was performed following establishment of a 20G peripheral venous line. Epidural analgesia was achieved using 0.5% ropivacaine (sensory block T4-T12). Sedation was maintained with intravenous ciprofol to achieve a Narcotrend index of 40-60, while patients maintained spontaneous respiration (15-30 breaths/min) with facemask oxygen at 6 L/min, SpO_2_ >95%, ETCO_2_ <80 mmHg, and tidal volume ≥5 mL/kg.

### Camera point location

**Figure 2:**
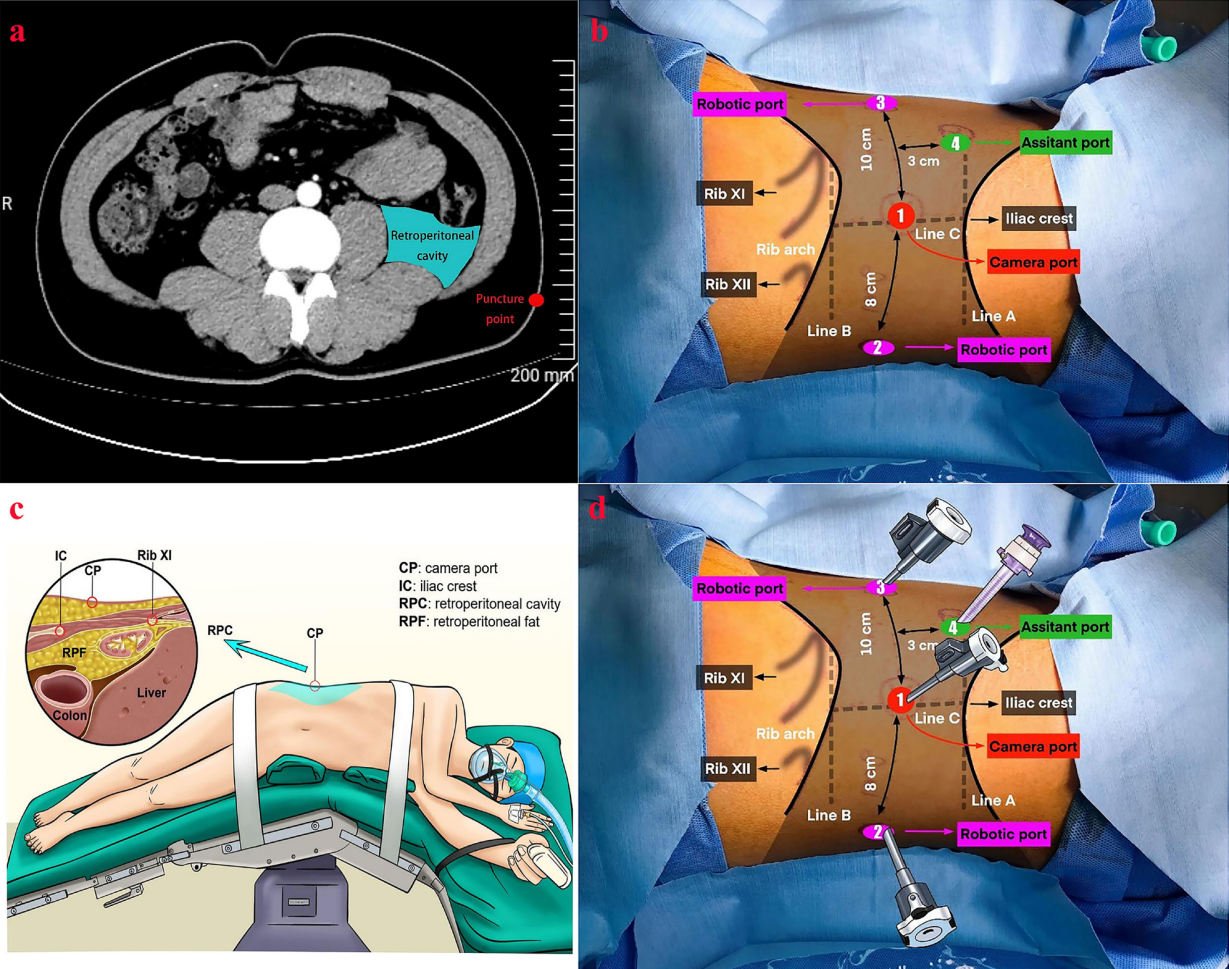
Procedures. 2a) Retroperitoneal cavity and puncture point; 2b) Trocars location; 2c) Patient position; 2d), Port placement.

Preoperative CT was used to evaluate volume of the retroperitoneal cavity and determine the ideal camera puncture site by measuring the thickness of subcutaneous fat and muscle (Figure-2a). Initially, the highest point of the iliac crest was marked to create a sagittal reference axis (Line A). Next, the costal margin between the 11th and 12th ribs was identified to draw a second sagittal axis (Line B). From the highest point on the iliac crest, a perpendicular line (Line C) was drawn to intersect line AB. The midpoint of line C was designated as the camera port puncture site (Figure-2b).

### Patient positioning and surgical procedure

Following anesthesia induction, urinary catheterization was omitted in the TLRRPN group and routinely applied in the TRRPN group. All patients were placed in a lateral decubitus position with the tumor side up. The coronal plane was maintained perpendicular to the operating table. Both arms were positioned forward, and the table was flexed to enlarge the retroperitoneal space between the iliac crest and costal margin (Figure-2c).

A 1.2 cm vertical incision was made at the midpoint of Line C. The depth of puncture was manually controlled, guided by subcutaneous and muscle thickness measurements from preoperative CT. Once a "breakthrough" sensation indicated entry into the posterior abdominal cavity, the surgeon inserted an index finger and gently rotated 180° along the abdominal wall to develop a retroperitoneal space between the posterior renal fascia and transversalis fascia (see video). During this maneuver, tactile confirmation of the smooth transversalis fascia, the 12th rib, and the psoas muscle body was required.

The first 8 mm robotic port was placed 8 cm posterior to the camera port in the same sagittal plane. The second 8 mm port was inserted 10-12 cm anterior to the first. A 12 mm assistant port was positioned 3-5 cm below the midpoint between the camera and anterior robotic ports. All ports were inserted bluntly under tactile guidance with the index finger placed through the camera incision to ensure safe passage into the retroperitoneal space (Figure-2d and see video). Following these highly simplified steps, the surgeon can complete the trocar placement within one minute.

The da Vinci Xi System™ was docked dorsally. Bipolar forceps were positioned in the left robotic arm and monopolar curved scissors in the right, with pneumoperitoneum maintained at 12 mmHg. After incising the lateral fascia, the perirenal fat was exposed, enlarging the working cavity. Retroperitoneal fat was cleared as required. Using the psoas muscle as a reference, we identified the perirenal fat and traced the renal artery pulsation to locate the renal hilum, after which standard RPN procedures were performed (see video). The specimen was extracted via the camera port with meticulous hemostasis.

In TLRRPN cases, no drainage tube was placed. However, if intraoperative suturing is deemed unsatisfactory, placement of an abdominal drainage tube is permitted. In contrast, a drainage tube was routinely inserted in the TRRPN group.

### Postoperative management

Hemoglobin levels were monitored via complete blood counts immediately after surgery, at 9 hours, and on postoperative day 1. Absence of a significant decline suggested a low risk of active bleeding. TLRRPN patients were encouraged to resume oral intake and ambulation within 1 hour postoperatively. No routine intravenous fluids or antibiotics were used, and only minimal oral analgesics were provided as needed. In contrast, TRRPN patients received patient-controlled analgesia, intravenous hydration until return of gastrointestinal function, and progressive mobilization. The urinary catheter, central venous catheter, and drainage tube were removed sequentially before discharge. Postoperative complications were graded using the Clavien-Dindo system, and pain intensity was assessed using the Visual Analog Scale (VAS).

## Statistical Analysis

PSM using logistic regression was applied to minimize confounding and balance baseline variables (age, sex, BMI, and RENAL score). A 1:1 nearest-neighbor match was performed with a caliper of 0.05 on the logit scale.

Continuous variables were compared using two-tailed Student's t-tests, while categorical variables were analyzed using chi-square or Fisher's exact tests, as appropriate. A two-sided p-value < 0.05 was considered statistically significant. Statistical analyses were performed using SPSS software (Version 27.0; IBM Corp, Armonk, NY, USA).

## RESULTS

**Figure 3:**
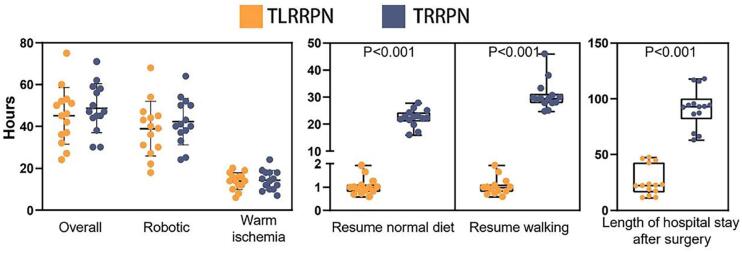
Intraoperative and postoperative outcomes. TLRRPN, tubeless retroperitoneal robot-assisted partial nephrectomy; TRRPN, traditional retroperitoneal robot-assisted partial nephrectomy.

Twenty-eight patients were included in the final analysis (TLRRPN, n = 14; TRRPN, n = 14), with a median follow-up of 303 days (range: 195-540 days). The median age was 48.86 ± 13.39 years, the median BMI was 24.97 ± 3.76 kg/m^2^, and the median RENAL score was 7.64 ± 1.44. Baseline characteristics did not differ significantly between groups (Table-1). There were no anesthesia- or surgery-related deaths, no conversions to open surgery, and all patients achieved R0 resection. Overall operative time, robotic operating time, warm ischemia time, and estimated blood loss were comparable between the TLRRPN and TRRPN groups (Figure-3 and Table-2).

**Table 1:** Baseline preoperative characters of study after 1:1 propensity score match.

Characteristic		Total (n=28)	TLRRPN (n=14)	TRRPN (n=14)	P value
**Median Age**		48.86±13.39	48.57±12.43	49.14±14.75	0.913
**Gender**					
	Male	24 (85.7%)	12 (85.7%)	12 (85.7%)	1.000
	Female	4 (14.3%)	2 (14.3%)	2 (14.3%)	
**BMI(Kg/m^2^)**		24.97±3.76	25.24±3.68	24.71±3.97	0.716
**18.5-23.9**		13 (46.4%)	7 (50.0%)	6 (42.9%)	0.705
	≥24	15 (53.6%)	7 (50.0%)	8 (57.1%)	
**Tumor laterality**					
	Left	14 (50.0%)	6 (42.9%)	8 (57.1%)	0.450
	Right	14 (50.0%)	8 (57.1%)	6 (42.9%)	
**Tumor positions**					
	Anterior	17 (60.7%)	10 (71.4%)	7 (50.0%)	0.246
	Posterior	11 (29.3%)	4 (28.6%)	7 (50.0%)	
**Tumor size (cm)**		3.02±0.68	3.04±0.61	3.00±0.76	0.893
**RENAL score**		7.64±1.44	7.64±1.49	7.64±1.44	1.000
**ASA status class**					
	I	23 (82.1%)	11 (78.6%)	12 (85.7%)	1.000
	II	5 (17.9%)	3 (21.4%)	2 (14.3%)	
**Abdominal surgery history**					
	No	25 (89.3%)	13 (92.9%)	12 (85.7%)	1.000
	Yes	3 (10.7%)	1 (7.1%)	2 (14.3%)	
**Preoperative eGFR (mL/min/1.73m^2^)**		101.58±31.80	107.61±36.44	95.56±26.36	0.326

Qualitative Variables are given as number (%); Quantitative Variables are given as median ± SD.

TLRRPN = tubeless robot-assisted partial nephrectomy; TRRPN = traditional retroperitoneal robot-assisted partial nephrectomy; BMI = body mass index; RENAL = R (radius), E (exophytic/ endophytic), N (nearness), A (anterior), L (location); ASA = American Society of Anesthesiologist; eGFR = estimated glomerular filtration rate.

**Table 2:** Comparison of perioperative outcomes between TLRRPN and TRRPN.

Characteristic		Total (n=28)	TLRRPN (n=14)	TRRPN (n=14)	P value
**Overall operative time (min)**		46.86±12.58	45.00±13.55	48.71±11.73	0.445
**Robotic operative time (min)**		40.54±12.04	38.86±13.11	42.21±11.09	0.471
**Trocar placement and robot docking time (min)**		3.39±0.49	3.36±0.49	3.43±0.51	0.712
**Opening and closing abdomen time (min)**		2.93±0.60	2.79±0.42	3.07±0.73	0.217
**Estimated blood loss (ml)**		26.96±13.69	29.29±14.39	24.64±13.07	0.380
**Warm ischemia time (min)**		14.14±4.30	13.93±4.04	14.36±4.70	0.798
**Transfusion required**		None	None	None	1.000
**Conversion to open**		None	None	None	1.000
**Conversion to radical nephrectomy**		None	None	None	1.000
**Peritoneal rupture**		None	None	None	1.000
**Time to begin walking after surgery (h)**		15.79±15.51	1.03±0.36	30.55±5.50	<0.001
**Time to resume normal diet after surgery (h)**		11.61±11.00	1.03±0.36	22.20±3.18	<0.001
**Length of hospital stay after surgery (h)**		58.72±36.61	26.00±13.11	91.43±17.52	<0.001
**1d postoperative eGFR (ml/min/1.73m^2^)**		93.11±26.90	97.34±32.78	88.87±19.74	0.415
**Pathology**					0.677
	Clear cell	20 (71.4%)	9 (64.3%)	11 (78.6%)	
	Chromophobe	3 (10.7%)	2 (14.3%)	1 (7.1%)	
	SDHRCC	1 (3.6%)	1 (7.1%)	0 (0.0%)	
	Benign	4 (14.3%)	2 (14.3%)	2 (14.3%)	
**Pathological stage**					0.593
	T1a	23 (82.1%)	12 (85.7%)	11 (78.6%)	
	T3a	1 (3.6%)	0 (0.0%)	1 (7.1%)	
	Benign	4 (14.3%)	2 (14.3%)	2 (14.3%)	
**Positive surgical margin**		All negative	All negative	All negative	1.000
**Hospitality cost (thousands CNY)**		63.93±6.48	58.14±1.56	69.71±3.56	**<0.001**
**Postoperative symptoms and complications**		Total (n=28)	TLRRPN (n=14)	TRRPN (n=14)	P value
**Anesthesia-related symptoms**				
Dizziness		14 (50.0 %)	0 (0.0%)	14 (100%)	<0.001
Nausea and vomiting		12 (42.8 %)	0 (0.0%)	12 (85.7%)	<0.001
Hoarseness		1 (3.5%)	0 (0.0%)	1 (7.1%)	0.500
Cough		14 (50%)	0 (0.0%)	14 (100%)	<0.001
Hemoptysis		1 (3.5%)	0 (0.0%)	1 (7.1%)	0.500
Sore throat		6 (21.4%)	0 (0.0%)	6 (42.8%)	0.008
**Other symptoms**				
Fever		2 (7.1%)	0 (0.0%)	2 (14.2%)	0.481
Drainage tube associated pain		9 (32.1%)	0 (0.0%)	9 (64.2%)	<0.001
Urethral discomfort		13 (46.4%)	0 (0.0%)	13 (92.8%)	<0.001
**Clavien-Dindo Complication**				
Clavien I		6 (21.4%)	0 (0.0%)	6 (42.8%)	0.008
**VAS pain score**		3.18±1.15	2.14±0.36	4.21±0.57	<0.001

Note: Qualitative Variables are given as number (%); Quantitative Variables are given as median ± SD.

Abbreviation: TLRRPN, tubeless robot-assisted partial nephrectomy; TRRPN, traditional retroperitoneal robot-assisted partial nephrectomy; eGFR, estimated glomerular filtration rate; SDHRCC, succinate dehydrogenase renal cell cancer; CNY, Chinese Yuan; VAS, visual analog scale.

Postoperative recovery was markedly faster in the TLRRPN group. Time to oral intake (1.03 ± 0.36 hours vs. 22.20 ± 3.18 hours, p < 0.001) and time to ambulation (1.03 ± 0.36 hours vs. 30.55 ± 5.50 hours, p < 0.001) were markedly shorter in the TLRRPN group. Median postoperative length of stay was notably reduced (26.00 ± 13.11 hours vs. 91.43 ± 17.52 hours; p < 0.001), as were hospitalization costs (58,500 CNY vs. 69,000 CNY; p < 0.001) (Figure 3 and Table-2). The tumor margins for all patients were negative. Pathological analysis showed that 82.1% (23/28) of tumors were pT1a, while 1 TRRPN patient had pT3a disease and is under close follow-up without recurrence (Table-2).

In the TLRRPN group, no anesthesia- or surgery-related symptoms were reported (Table-2). In contrast, the TRRPN group reported dizziness in 14 patients (p < 0.001), nausea/vomiting in 12 patients (p < 0.001), hoarseness in 1 patient, productive cough in 14 patients (p < 0.001), hemoptysis in 1 patient, and sore throat in 6 patients (p = 0.008). Furthermore, 2 patients from the TRRPN group developed postoperative fever, 9 reported drain-associated pain (p < 0.001), and 13 experienced urethral discomfort (p < 0.001). According to the Clavien-Dindo classification system, 6 patients within the TRRPN group experienced Grade I complications (p = 0.008). Pain scores assessed using the VAS were significantly lower in the TLRRPN group (2.14 ± 0.36 vs. 4.21 ± 0.57; p < 0.001) (Table-2).

## DISCUSSION

The progression from OPN to RRPN represents the continuous pursuit of greater surgical precision, reduced trauma, and enhanced perioperative recovery (1-4, 20, 21). In addition to oncological efficacy, contemporary practice increasingly emphasizes patient-centered care, including individualized treatment and improved perioperative experience. Tubeless pulmonary surgery has demonstrated safety, feasibility, and rapid recovery, and has therefore gained widespread adoption globally (22-24). Anatomically, the retroperitoneal cavity is isolated from intraperitoneal organs, allowing CO_2_ insufflation to be contained without significant diaphragmatic or respiratory compromise (25-27). This enables maintenance of spontaneous ventilation using TLRRPN and avoids the need for tracheal intubation, muscle relaxants, and excessive sedatives. Moreover, by eliminating bowel manipulation, the physiological stress of surgery is reduced. Robotic precision contributes to minimal tissue injury, accurate suturing, and reliable hemostasis, obviating the need for retroperitoneal drainage. Within this controlled operative environment, all procedures were successfully completed within 90 minutes, without urinary catheterization. Fluid requirements remained low, and central venous access was unnecessary.

This study presents preliminary findings of TLRRPN, an innovative technique that avoids tracheal intubation, central venous access, urinary catheterization, and abdominal drainage. Our findings demonstrate clear advantages over TRRPN. Compared to TRRPN, patients undergoing TLRRPN were able to resume oral intake and ambulation within 1 hour postoperatively, effectively reducing tube-related discomfort and accelerating functional recovery. The perioperative experience closely resembled that of painless gastroscopy, enabling discharge within 24 hours. Furthermore, this approach significantly reduced overall hospitalization costs.

From the patient's perspective, TLRRPN significantly alleviates discomfort and reduces the risk of complications. Avoiding tracheal intubation, muscle relaxants, and excessive sedatives minimizes postoperative symptoms, such as sore throat, cough, dizziness, and sputum retention. Similarly, omission of central venous and urinary catheters reduces the incidence of infection, thrombosis, and lower urinary tract irritation, while the absence of a drain leads to less postoperative pain. Notably, the simplified anesthetic regimen also helps preserve gastrointestinal function, enabling rapid resumption of oral intake and early ambulation.

From the surgeon's standpoint, TLRRPN focuses on atraumatic peritoneal handling to prevent intraoperative escalation. Adopting a three-arm robotic configuration enhances maneuverability in the retroperitoneal space while significantly reducing the risk of peritoneal injury compared to conventional four-arm systems. Tumor exposure is optimized by targeted incision of the lateral conal fascia and selective insufflation, allowing focused dissection without extensive mobilization. Resection and renorrhaphy follow standard RPN protocols, and any peritoneal breach is promptly closed with clips to maintain operative integrity. In our clinical practice, a pneumoperitoneum pressure of 12 mmHg is sufficient to maintain the surgical space. If peritoneal rupture is detected intraoperatively, it compromises the surgical space and allows pneumoperitoneum to enter the abdominal cavity, impairing diaphragmatic respiration. Prompt repair is therefore essential. Abdominal gas can be evacuated with an aspirator, and the defect may be closed using clips or barbed sutures.

Anesthesiologists are integral to the safe and effective implementation of TLRRPN. Preoperative preparation involves placing an epidural catheter and securing peripheral venous access. Throughout the procedure, continuous monitoring of hemodynamic and respiratory parameters is essential. With the robotic tower positioned posteriorly, adequate anterior workspace remains for anesthesiologists to manage the airway and ventilation.

In patients at increased risk of collecting system rupture or postoperative hematuria, selective postoperative urinary catheterization may be warranted. If required, intraoperative conversion to a traditional airway or vascular access is feasible: ultrasound-guided central venous catheterization in the lateral position can usually be accomplished within 3 to 5 minutes, and video laryngoscope–assisted tracheal intubation requires a similar time frame. Therefore, TLRRPN can be safely and rapidly converted to TRRPN when necessary.

Owing to the robot's high-precision suturing capability, the risk of postoperative bleeding following early-stage renal tumor surgery is exceedingly low. Consequently, we routinely omit placement of an abdominal drainage tube during the procedure. Ronney Abaza et al. have demonstrated the safety and feasibility of omitting routine abdominal drainage following partial nephrectomy for early-stage renal tumors (28). Nevertheless, selective use of an abdominal drainage tube remains an option. Specifically, we might place a drain in cases involving deeply located tumors, large surgical wounds, substantial perirenal fat, or anticipated significant postoperative fluid accumulation, as judged intraoperatively.

TLRRPN necessitates a highly experienced surgical team to ensure both patient safety and procedural success. Although TLRRPN presents considerable technical challenges, we carefully selected patients with localized, early-stage renal tumors and rigorously standardized each step of the highly simplified, reproducible surgical protocol. Adherence to this protocol enabled successful completion of TLRRPN in our cohort; we hope that this approach will herald a new, tubeless era in the management of renal tumors.

This article presents the first reported description of a tubeless surgical approach for the management of early-stage renal tumors. It further validates both the safety of tubeless surgery under pneumoperitoneum and its feasibility for routine clinical implementation. By minimizing or eliminating the use of tubes, this technique fundamentally mitigates tube-related complications and patient discomfort. Patients may resume normal activities and oral intake as early as one hour postoperatively. However, this surgical strategy is highly selective and is not indicated for patients with complex renal tumor.

Despite the promising results, our study has several limitations. From April 2025 to the present, we have successfully performed over 90 cases of TLRRPN; however, only 14 cases have achieved adequate follow-up duration. As such, our assessment primarily reflects feasibility and perioperative safety, while long-term oncologic outcomes remain to be determined. The sample size is relatively small, and the procedure is currently applicable only to carefully selected patients with early-stage renal tumors (RENAL score ≤9) and expected operative times under 90 minutes. Successful implementation also necessitates an experienced surgical, anesthetic, and nursing team, thereby confining its use to high-volume centers. Finally, data were collected retrospectively, and the small sample size may introduce potential selection bias. Therefore, larger prospective clinical studies are needed to validate the long-term safety, oncologic efficacy, and broader applicability of this novel approach.

## CONCLUSIONS

TLRRPN is a safe and effective surgical option for carefully selected patients with early-stage renal tumors. By minimizing perioperative invasiveness, it improves patient comfort, accelerates recovery, and reduces overall hospitalization costs. This technique represents a promising step toward more refined, patient-centered minimally invasive surgery.

## Data Availability

All data generated or analysed during this study are included in this published article

## References

[1] Kim SB, Williams SB, Cheng SC, Sanda MG, Wagner AA (2012). Evaluation of patient-reported quality-of-life outcomes after renal surgery. Urology.

[2] Yu HY, Hevelone ND, Lipsitz SR, Kowalczyk KJ, Hu JC (2012). Use, costs and comparative effectiveness of robotic assisted, laparoscopic and open urological surgery. J Urol.

[3] Maurice MJ, Kaouk JH, Ramirez D, Bhayani SB, Allaf ME, Rogers CG (2017). Robotic partial nephrectomy for posterior tumors through a retroperitoneal approach offers decreased length of stay compared with the transperitoneal approach: a propensity-matched analysis. J Endourol.

[4] Boga MS, Sonmez MG, Karamik K, Ozsoy C, Aydin A, Savas M (2021). Long-term outcomes of minimally invasive surgeries in partial nephrectomy. Robot or laparoscopy?. Int J Clin Pract.

[5] Socarras MR, Elbers JR, Rivas JG, Autran AM, Esperto F, Tortolero L (2021). Retroperitoneal robot-assisted partial nephrectomy (RRAPN): surgical technique and review. Curr Urol Rep.

[6] Carbonara U, Crocerossa F, Campi R, Veccia A, Cacciamani GE, Amparore D (2022). Retroperitoneal robot-assisted partial nephrectomy: a systematic review and pooled analysis of comparative outcomes. Eur Urol Open Sci.

[7] Orsini A, Lasorsa F, Bignante G, Yoon J, Dymanus KA, Cherullo EE (2024). Single port robotic nephrectomy via lower anterior retroperitoneal approach: feasible, safe and effective option in surgically complex patients. Int Braz J Urol.

[8] El-Boghdadly K, Bailey CR, Wiles MD (2016). Postoperative sore throat: a systematic review. Anaesthesia.

[9] van Esch BF, Stegeman I, Smit AL (2017). Comparison of laryngeal mask airway vs tracheal intubation: a systematic review on airway complications. J Clin Anesth.

[10] Coppadoro A, Bellani G, Foti G (2019). Non-pharmacological interventions to prevent ventilator-associated pneumonia: a literature review. Respir Care.

[11] Yang SS, Wang NN, Postonogova T, Yang GJ, McGillion M, Beique F (2020). Intravenous lidocaine to prevent postoperative airway complications in adults: a systematic review and meta-analysis. Br J Anaesth.

[12] Boulton AJ, Smith E, Yasin A, Moreton J, Mendonca C (2024). Tracheal tube introducer-associated airway trauma: a systematic review. Anaesthesia.

[13] Scruggs-Wodkowski E, Kidder I, Meddings J, Patel PK (2024). Urinary catheter-associated infections. Infect Dis Clin North Am.

[14] Li S, Li P, Wang R, Li H (2022). Different interventions for preventing postoperative catheter-related bladder discomfort: a systematic review and meta-analysis. Eur J Clin Pharmacol.

[15] Wilson M (2008). Causes and management of indwelling urinary catheter-related pain. Br J Nurs.

[16] Teja B, Bosch NA, Diep C, Pereira TV, Mauricio P, Sklar MC (2024). Complication rates of central venous catheters: a systematic review and meta-analysis. JAMA Intern Med.

[17] Geerts W (2014). Central venous catheter-related thrombosis. Hematology Am Soc Hematol Educ Program.

[18] Gundogan E, Kayaalp C, Aktas A, Saglam K, Sansal M, Gokler C (2018). Influence of drain placement on postoperative pain following laparoscopic Roux-en-Y gastric bypass for morbid obesity: randomized controlled trial. Obes Surg.

[19] George NM, Chitrambalam TG, Christopher PJ, Marlecha M, Selvamuthukumaran S (2023). To drain or not to drain following thyroidectomy: a prospective, randomized study. Saudi Med J.

[20] Zhang Z, Li Z, Xu W, Wang X, Zhu S, Dong J (2024). Robot-assisted radical nephroureterectomy using the Kangduo Surgical Robot-01 system versus the da Vinci system: a multicenter prospective randomized controlled trial. Int Braz J Urol.

[21] Duarte DMJ, Brum PW, Berger M, Kowalski A, Silva BN, Berger AK (2026). Robot-assisted partial nephrectomy with and without mixed reality - REALITATEM study. Int Braz J Urol.

[22] Lirio F, Galvez C, Bolufer S, Corcoles JM, Gonzalez-Rivas D (2018). Tubeless major pulmonary resections. J Thorac Dis.

[23] Li S, Jiang L, Ang KL, Chen H, Dong Q, Yang H (2017). New tubeless video-assisted thoracoscopic surgery for small pulmonary nodules. Eur J Cardiothorac Surg.

[24] He J, Liang H, Wang W, Akopov A, Aiolfi A, Ang KL (2021). Tubeless video-assisted thoracic surgery for pulmonary ground-glass nodules: expert consensus and protocol (Guangzhou). Transl Lung Cancer Res.

[25] Hughes-Hallett A, Patki P, Patel N, Barber NJ, Sullivan M, Thilagarajah R (2013). Robot-assisted partial nephrectomy: a comparison of the transperitoneal and retroperitoneal approaches. J Endourol.

[26] Garg M, Singh V, Sinha RJ, Sharma P (2014). Prospective randomized comparison of transperitoneal vs retroperitoneal laparoscopic simple nephrectomy. Urology.

[27] Pellegrino AA, Chen G, Morgantini L, Calvo RS, Crivellaro S (2023). Simplifying retroperitoneal robotic single-port surgery: novel supine anterior retroperitoneal access. Eur Urol.

[28] Abaza R, Prall D (2013). Drain placement can be safely omitted after the majority of robotic partial nephrectomies. J Urol.

