# Supplementary material for: Tubeless Retroperitoneal Robot-assisted Partial Nephrectomy: An Innovative Approach to Treat Early Renal Tumor
Source: Int Braz J Urol. 2026 Apr 25;52(4):e20260042. doi: 10.1590/S1677-5538.IBJU.2026.0042 (PMC13400067; doi:10.1590/S1677-5538.IBJU.2026.0042)
Supplement: Supplementary Information [file 1677-6119-ibju-52-04-e20260042-suppl01.pdf]

APPENDIX

Supplementary Table. Preoperative characters of study before 1:1 propensity score match.

Characteristic	Total (n=78)	TLRRPN (n=14)	TRRPN (n=64)	P value
**Median Age**	51.86±1.66	48.57±12.43	52.58±15.14	0.359
**Gender**				0.211
Male	55 (70.5%)	12 (85.7%)	43 (67.2%)	
Female	23 (29.5%)	2 (14.3%)	21 (32.8%)	
**BMI(Kg/m²)**	24.2±0.3	25.2±3.6	24.0±3.4	0.231
18.5-23.9	38 (48.7%)	7 (50.0%)	31 (48.4%)	1.000
≥24	40 (51.3%)	7 (50.0%)	33 (51.6%)	
**Tumor laterality**				0.486
Left	40 (51.3%)	6 (42.9%)	34 (53.1%)	
Right	38 (48.7%)	8 (57.1%)	30 (46.9%)	
**Tumor positions**				0.015
Anterior	33 (42.3%)	10 (71.4%)	23 (35.9%)	
Posterior	45 (57.7%)	4 (28.6%)	41 (64.1%)	
**Tumor size (cm)**	3.10±0.96	3.04±0.61	3.11±0.90	0.775
**RENAL score**	7.60±0.17	7.64±1.49	7.59±1.50	0.912
**ASA status class**				0.554
I	52 (66.7%)	11 (78.6%)	41 (64.0%)	
II	24 (32.1%)	3 (21.4%)	22 (34.4%)	
III	1 (1.2%)	0 (0.0%)	1 (1.6%)	
**Abdominal surgery history**				0.678
No	67 (85.9%)	13 (92.9%)	54 (84.4%)	
Yes	11 (14.1%)	1 (7.1%)	10 (15.6%)	
**Preoperative eGFR (mL/min/1.73m²)**	96.17±3.92	107.61±36.44	93.67±34.02	0.174

Qualitative Variables are given as number (%); Quantitative Variables are given as median ± SD.
TLRRPN, tubeless robot-assisted partial nephrectomy; TRRPN, traditional retroperitoneal robot-assisted partial nephrectomy; BMI, body mass index; RENAL, R (radius), E (exophytic/ endophytic), N (nearness), A (anterior), L (location); ASA, American Society of Anesthesiologist; eGFR, estimated glomerular filtration rate.
